# A Survey of Policies and Local Ordinances Supporting Physical Activity in Hawaii Counties

**Published:** 2007-12-15

**Authors:** Katie M. Heinrich, Courtney B. Johnson, Yuka Jokura, Blythe Nett, Jay E Maddock

**Affiliations:** University of Hawaii at Manoa, Department of Public Health Sciences; University of Hawaii at Manoa, Honolulu, Hawaii; University of Hawaii at Manoa, Honolulu, Hawaii; University of Hawaii at Manoa, Honolulu, Hawaii; University of Hawaii at Manoa, Honolulu, Hawaii

## Abstract

**Background:**

Features of the built environment that influence physical activity behavior characterize Active Community Environments.

**Context:**

Whether Active Community Environments policies exist in the state of Hawaii's four counties is unknown. The purpose of this study was to provide a baseline assessment of these policies in Hawaii.

**Methods:**

A survey assessing policies in six domains (i.e., sidewalks, bike lanes, greenways, recreational facilities, commercial buildings, and shared-use paths) was completed by employees of Hawaii planning departments.

**Consequences:**

Honolulu County had the most policies (n = 13), followed by Maui County (n = 6), Kauai County (n = 2), and Hawaii County (n = 1). Written policies were most prevalent in Honolulu County (n = 15), followed by Kauai County (n = 14), Hawaii County, (n = 4), and Maui County (n = 3). Sidewalk policies were reported for Honolulu County, Maui County (no written policies were found for Maui County), and Kauai County. Bike lane and greenway policies were found for Honolulu County (reported and written) and Kauai County (written). Recreation facility and pedestrian shared-use path policies existed for all counties, although only Honolulu and Kauai counties had written policies for commercial buildings (Maui County reported having policies). Few policies directly addressed physical activity promotion.

**Interpretation:**

The most populous county, Honolulu, had the most policies in place, although discrepancies existed between reported and written policies. This baseline measure of physical activity–related policies will help focus efforts of county coalitions to increase opportunities for physical activity. Additional policies should be tracked with population behavior surveillance.

## Background

Physical inactivity is a major public health concern in the United States and contributes to the obesity epidemic (1). A slight increase in physical activity levels among inactive people has a major impact on the improvement of public health (2). The built environment influences physical activity of community residents by providing visual cues and opportunities for activity (3).

Moderate physical activity levels are linked to how communities are designed (4). For example, residents of neighborhoods with mixed uses (i.e., neighborhoods that include homes as well as stores, parks, offices, or other land uses) (5) and residents of neighborhoods with exercise facilities tend to have higher physical activity levels than do residents of neighborhoods that are not designated as mixed-use or that do not have exercise facilities (3,6). Active Community Environments (ACEs) are environments with characteristics that promote physical activity, such as public access to facilities, streets with sidewalks, and increased housing density (5).

Public policy is an essential part of a comprehensive approach to community health promotion (7), and public health programs have begun focusing on how environmental influences create opportunities for and remove barriers to physical activity (2,8,9). The behavior of anyone who comes into contact with an environment that has such policies in place may be influenced by that environment (9).

A policy-level measurement of built-environment factors of ACEs was developed by the Centers for Disease Control and Prevention (CDC) and tested in Utah (10). The identification of ACEs policies determined baseline policies already in place that could help direct future physical activity interventions in the state. The study also helped identify the individuals who were in charge of ACEs-related policies (10).

A study in New Zealand found that policy development and planning needed to avoid increasing inequalities, such as causing deprived and isolated areas to end up with fewer resources (11). In metropolitan Columbus, Ohio, most areas did not plan for pedestrians, and a wide variance was found between existing sidewalk policies, although up to 7% of the area's population reported walking to work (12). Instead, legislation that alters the built environment to encourage physical activity could include implementing building codes that would make stair locations visible and create an appealing alternative to elevator or escalator use (2). Policies could require developers to plan areas with more available parks and exercise facilities. Limits could be imposed on vehicular transportation to create attractive and safe pedestrian and bicycle paths and trails (2). Planners can help individuals meet physical activity recommendations by designing pedestrian-friendly neighborhoods (4).

## Context

In 2000, the state of Hawaii devoted a large percentage of its tobacco settlement dollars to fund health promotion efforts, including physical activity promotion. There are four main counties in Hawaii: 1) Kauai, 2) Honolulu, 3) Maui, and 4) Hawaii. The U.S. Census Bureau estimates that in 2005, 71% (n = 905,266) of the state's population resided in Honolulu County, followed by Hawaii County (13%, n = 167,293), Maui County (11%, n = 139,884), and Kauai County (5%, n = 62,640) (13). The state is developing county coalitions for the promotion of physical activity and nutrition. Currently, no measurement of physical activity-related policies exists. Before encouraging the development of physical activity-related policies for the built environment in the state of Hawaii, it is important to determine what county policies are in place. The purpose of this study was to provide a baseline assessment of the existing policies and ordinances related to ACEs in the state of Hawaii. We expected to find more policies in existence in Honolulu County than any of the other three counties, since it comprises 71% of the state's population.

## Methods

### Instruments

We used the instrument developed by Librett, Yore, and Schmid (10) following a framework developed by DeVellis (14) to measure characteristics associated with ACEs. This 15-question survey assessed counties’ policies and ordinances related to six domains identified by CDC as being important for ACEs (10): 1) sidewalks, 2) bike lanes, 3) greenways, 4) recreational facilities, 5) commercial buildings, and 6) shared-use paths. A checklist was given for respondents to indicate whether policies or ordinances called for sidewalks, bike lanes, greenways, and recreation facilities in new, redeveloped, and mixed-use communities. Regarding commercial buildings, one question was asked about an ordinance requiring new commercial buildings and site plans to include elements that would encourage physical activity (e.g., pedestrian walkways, well-lit stairways, sidewalks). Regarding shared-use paths, one question asked about ordinances requiring building paths dedicated for different types of pedestrians (e.g., joggers, dog walkers), and one question asked about policies for building shared-use paths in easements in the county’s master plan. The percentage of the population that reported walking to work was calculated from the 2000 U.S. Census.

### Procedure

In the spring of 2006, we mailed a survey and cover letter explaining the study to employees from planning and permitting, parks and recreation, and public works departments of the four Hawaii counties. These departments were oversampled (i.e., 10 potential participants from four counties were contacted) to ensure a response from each of the four counties. Five surveys were returned, one each representing Kauai, Honolulu, and Hawaii and two representing Maui. (The two respondents from Maui provided identical answers.) All five participants were from their county's Department of Planning and Permitting, although one individual had a dual appointment with the Department of Parks and Recreation. Checkmarks for the existence of a policy or ordinance for each category were coded as *yes* and unchecked categories were coded as *no*. All data were entered by a graduate student into an SPSS database (SPSS 14.0, Chicago, Illinois). Frequencies and percentages were calculated to determine differences by county. To verify reported policies, we made follow-up contacts with each county to obtain the specific policy and ordinance language. The wording of the policies was analyzed for relevant terms and phrases (e.g., "promoting walking and bicycling"), and the policies that were written specifically to promote physical activity were identified.

## Consequences

The existence of ACEs policies and ordinances varied greatly among counties. As expected, Honolulu County reported the most policies in place at 13 (87%). Of the possible 15 policies, Hawaii County reported only one (7%) policy, Kauai County reported two (13%), and Maui County six (40%). Analysis of written policies found evidence of all 15 (100%) policies for Honolulu County, four (27%) policies for Hawaii County, 14 (93%) policies for Kauai County, and four (33%) policies for Maui County (Figure).

**Figure:**
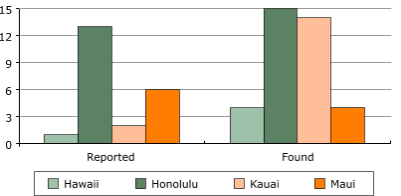
Number of Active Community Environment Ordinances by County, Hawaii, Spring 2006

**Table:** 

Of those people who reported working, the largest percentage who walked to work was found in Honolulu County at 5.6% (23,022/412,250) and ranged as low as 1.9% (500/26,183) in Kauai County. Sidewalk ordinances were the most common ordinances in place, with Maui, Kauai, and Honolulu counties reporting ordinances for new and redeveloped communities. Maui and Honolulu counties also reported having sidewalk ordinances for mixed-use communities. These reports of sidewalk ordinances in Honolulu and Kauai counties were substantiated by the written ordinances; however, no written sidewalk ordinances were found for Maui County.

Honolulu County was the only county that reported having ordinances for bike lanes and greenways for new, redeveloped, and mixed-use communities. An examination of the written policies showed that, in addition to the Honolulu County policies, Kauai County also addressed these areas (e.g., "Support funding to develop Kauai's bikeway system to provide for alternative means of transportation, recreation, and visitor activities [economic development].") (15).

Recreational facility policies were reported to exist for new communities in Hawaii, Honolulu, and Maui counties; for redeveloped communities in Honolulu County; and for mixed-use communities in Honolulu and Maui counties. In contrast, written policies were found for all three types of communities in each of the counties (e.g., "[Maui] County shall use the money received pursuant to this section for the purpose of providing parks and playgrounds for the use of purchasers or occupants of lots or units in the subdivision.") (16). Table 1 shows all reported and written ACEs policies for sidewalks, bikeways, greenways, and recreational facilities in each county.

Table 2 shows all reported and written ACEs policies for commercial buildings, shared-use paths for pedestrians, and shared-use paths designated in master plans. Only Honolulu and Maui reported having ordinances in place for commercial buildings. Examination of written policies verified that this was the case for Honolulu County. Although written commercial building ordinances were not found for Maui County, they did exist for Kauai County. No shared-use path ordinances were reported to exist in any county. However, written policies for shared-use paths for pedestrians were found in all counties and in the master plans for Honolulu and Kauai counties.

In-depth analysis of written policies revealed that few specifically addressed the promotion of physical activity (including recreational activities). Honolulu County, again, had the most policies with three, Hawaii and Kauai counties had two, and Maui County had one. These policies are displayed in Table 3.

## Interpretation

The existence of policies and ordinances related to the built environment and physical activity varies widely in the state of Hawaii. Honolulu County, where most of the state's population resides, had the most ordinances in place. It is encouraging that the most policies were reported in the county where most of the state's population would be affected. Through further analysis of written policies, we found that survey responses slightly underrepresented the existence of policies and ordinances that relate to promotion of physical activity. However, although Kauai County had written policies addressing all but one of the survey's categories, the survey response indicated only having sidewalk policies. The reason for this discrepancy is unclear.

Over 23,000 people reported walking to work in Honolulu County in 2000. Honolulu County is the only county in Hawaii that has urban areas, and it has the most comprehensive public bus transportation system. Honolulu County policies focus on improving the pedestrian environment: "Encourage walking and bicycling activities, especially walking to and from jobs, thus reducing automobile dependency and demands upon the transportation system" (17).

Hawaii County, the largest county by area, reported having only one policy in place for recreational facilities in new communities, although written policies also existed for recreational facilities in redeveloped and mixed-use communities, and a shared-use path policy existed for pedestrians. The extensive property development beginning to occur in Hawaii County provides an excellent opportunity to implement more policies related to physical activity promotion with the chance of influencing physical activity behavior and improving public health (2).

Although six policies were reported to exist in Maui County, only four written policies were found. It is possible that the other policies do exist, but we were unable to find them in written form.

The chance of being physically inactive increases 33% for residents without sidewalks in their neighborhoods (18). The presence of neighborhood sidewalks provides both a visual cue and an opportunity to participate in physical activity (3). Like more than 80% of cities studied in Utah (10), Honolulu, Kauai, and Maui counties are more likely to have sidewalks in their neighborhoods, which can help shape the behaviors of their residents (8).

Ordinances for sidewalks, bike lanes, greenways, and recreational facilities existed for mixed-use communities in Honolulu County, while Maui County had sidewalk and recreational facility ordinances for mixed-use communities, and Kauai County had bike lane, greenway, and recreational facility ordinances for mixed-use communities. This evidence of existing ordinances is encouraging, and the mere presence of mixed-use communities is important for physical activity, as residents of mixed-use communities have been shown to have higher physical activity levels (6).

Analysis of the written policies found provisions for recreational facilities in each of the counties. These provisions could lead to increased physical activity behaviors, as having more neighborhood facilities has been linked with higher physical activity levels (3,6). Additionally, although all four counties reported no ordinances or policies for shared-use paths, they were found to exist. These paths can help create transportation options that are appealing and practical for nonmotorized travel by providing visual cues for physical activity (3).

Overall, the distribution of ACEs policies for Hawaii's counties contrasts with policies found in Utah (10). Although there were few bike lane policies in Utah, 50% of Hawaii's counties have bike lane policies in place. Hawaii counties had 50% more policies related to greenways, commercial buildings, and recreation facilities than were found in Utah. The prevalence of shared-use paths was similar in both states (10).

Although this study determined which physical activity-related policies exist in Hawaii, future research could examine actual policy implementation and enforcement. Policy enforcement is important; for example, dangerous conditions for pedestrians can result from poorly maintained sidewalks (12). Future research could determine whether these counties always, sometimes, or never enforce their policies, and what policy enforcement entails.

When writing policy, policy makers tend to focus on practical land-use purposes and not on physical activity promotion. However, as Evans-Cowley reported (12), interest in the development of policies to promote pedestrian activity is increasing. Many local governments concerned with health issues are trying to address them through health-promoting design policies (12).

Future research could examine the environmental resources available for physical activity in each county to help identify where the need for ordinances and facilities is the greatest. Future research could also examine the consistency and overlap of policies related to physical activity at the state level in comparison with county-level and municipal-level policies. Additional research is needed to determine how the policies are implemented (e.g., when and to whom variances are granted) and enforced.

This study gives a baseline measure of all reported and written physical activity-related policies in Hawaii. County planning departments need to carefully plan for physical activity. These survey results suggest that adequate policies are in place in Honolulu County, where they have the best chance of affecting a large number of individuals. In the future, more effort should be placed on the neighbor islands of Maui and Hawaii, which are rapidly developing and where minimal ordinances exist, allowing the statewide coalition for nutrition and physical activity to focus efforts on new built-environment policies. Areas of focus could be deprived and isolated areas that may not have adequate opportunities for physical activity in their built environments (10). Residents of Hawaii and Maui counties could greatly benefit from the development of additional physical activity-related ordinances and policies. The implementation of any additional policies should be tracked along with their impact on population behavior.

## Figures and Tables

**Table 1:** Reported and Written Active Community Environment Policies for New, Redeveloped, and Mixed-Use Communities, Four Hawaii Counties, Spring 2006

County	Population That Walks to Work, %^a^ (n/N)	Sidewalks			Bike Lanes			Greenways			Recreational Facilities		

		N	R	M	N	R	M	N	R	M	N	R	M
Hawaii	3.0 (1,873/63,401)										X^b^Y^c^	Y	Y
Honolulu	5.6 (23,022/412,250)	XY	XY	XY	XY	XY	XY	XY	XY	XY	XY	XY	XY
Kauai	1.9 (500/26,183)	XY	XY	Y	Y	Y	Y	Y	Y	Y	Y	Y	Y
Maui	2.8 (1,739/61,262)	X	X	X							XY	Y	XY

N indicates new communities; R, redeveloped communities; M, mixed-use communities. Blank cells indicate that a policy did not exist for that category.

a Percentages calculated from total number of people who reported working.

b X = Policy was reported to exist by survey respondent.

c Y = Policy wording was found in analysis of existing policies.

**Table 2:** Reported and Written Active Community Environment Policies for Commercial Buildings and Shared-Use Paths, Four Hawaii Counties, Spring 2006

County	Commercial Buildings	Shared-Use Paths for Pedestrians	Shared-Use Paths in Master Plan
Hawaii		Y^a^	
Honolulu	X^b^Y	Y	Y
Kauai	Y		Y
Maui	X	Y	

Blank cells indicate that a policy did not exist for that category.

a Y = Policy wording was found in analysis of existing policies.

b X = Policy was reported to exist by survey respondent.

**Table 3:** Written Policies Promoting Physical Activity, by County and Policy Focus, Four Hawaii Counties, Spring 2006

County	Policy Category	Policy
Hawaii	Shared-use paths	Develop facilities and safe pathway systems for walking, jogging, and biking activities (Recreation Policy 12.3-o).
	Recreational facilities	Encourage combining schoolyards with county parks and allow school facilities for after school use by the community for recreational, cultural, and other compatible uses (Education Policy 10.2.2-b).
Honolulu	New commercial buildings or developments	Encourage walking and bicycling activities, especially walking to and from jobs, thus reducing automobile dependency and demands upon the transportation system (Revised Ordinances of Honolulu Sec. 24-1.4-g-1-B). Provide greater opportunities for variety in urban experiences for pedestrians (Revised Ordinances of Honolulu Sec. 24-1.4-g-1-D).
	Shared-use paths	Pedestrian corridors shall be provided in heavy traffic areas, such as in resort, commercial, and apartment districts. Such elements as shade trees and other plantings, street furniture, attractive building frontages, and other pedestrian-oriented elements shall be part of the design of pedestrian corridors. Pedestrian corridors shall be designed to be safe, minimize conflicts between people and vehicular movements, and shall be integrated with or provide access to open spaces (Revised Ordinances of Honolulu Sec. 14-1.4-c).
	Recreational facilities	State and County Parks and Recreation Sites. Preservation/Forest Areas. Areas of recreational value shall be of low-intensity use. When development prevents the establishment of mountain parks, streamside parks, or other upland recreational facilities, public access shall be made available to the resource. Points of access to hiking trails, hunting areas, swimming areas, and camping areas shall be established as provided under Ordinance No. 4311 (1974), "Public Access of Pedestrian Traffic to Shoreline and Mountain Areas" (Revised Ordinances of Honolulu Sec. 24-1.5-a-1-A).
Kauai	Bike lanes	Support funding to develop Kauai's bikeway system to provide for alternative means of transportation, recreation, and visitor activities (Policy 7.3.2).
	Recreational facilities	Provide additional beach park areas and ocean recreation facilities. Encourage alternative recreational activities on private lands (Policy 6.4.4.2).
Maui	Recreational facilities	The general purpose and intent of the park district ordinances are to preserve and manage lands for passive or active recreational activities by a system of parks suited to the varying recreational needs of the county, to provide parks which are of differing sizes and uses, and to implement the general plan and community plans of the county and the land use laws of the state (Policy 19.615.010).
